# Comparison of silver and molybdenum microfocus X-ray sources for single-crystal structure determination

**DOI:** 10.1107/S1600576714022985

**Published:** 2015-01-30

**Authors:** Lennard Krause, Regine Herbst-Irmer, George M. Sheldrick, Dietmar Stalke

**Affiliations:** a Institut für Anorganische Chemie, Georg-August Universität Göttingen, Tammannstrasse 4, 37077 Göttingen, Germany

**Keywords:** microfocus X-ray sources, single-crystal structure determination, absorption correction

## Abstract

The quality of diffraction data obtained using silver and molybdenum microsources has been compared for six model compounds with a wide range of absorption factors. The experiments were performed on two 30 W air-cooled Incoatec IµS microfocus sources with multilayer optics mounted on a Bruker D8 goniometer with a SMART APEX II CCD detector. All data were analysed, processed and refined using standard Bruker software. The results show that Ag *K*α radiation can be beneficial when heavy elements are involved. A numerical absorption correction based on the positions and indices of the crystal faces is shown to be of limited use for the highly focused microsource beams, presumably because the assumption that the crystal is completely bathed in a (top-hat profile) beam of uniform intensity is no longer valid. Fortunately the empirical corrections implemented in *SADABS*, although originally intended as a correction for absorption, also correct rather well for the variations in the effective volume of the crystal irradiated. In three of the cases studied (two Ag and one Mo) the final *SHELXL R*1 against all data after application of empirical corrections implemented in *SADABS* was below 1%. Since such corrections are designed to optimize the agreement of the intensities of equivalent reflections with different paths through the crystal but the same Bragg 2θ angles, a further correction is required for the 2θ dependence of the absorption. For this, *SADABS* uses the transmission factor of a spherical crystal with a user-defined value of μ*r* (where μ is the linear absorption coefficient and *r* is the effective radius of the crystal); the best results are obtained when *r* is biased towards the smallest crystal dimension. The results presented here suggest that the IUCr publication requirement that a numerical absorption correction must be applied for strongly absorbing crystals is in need of revision.

## Abstract 2

A detailed comparison of single-crystal diffraction data collected with Ag *K*α and Mo *K*α microsources (IµS) indicates that the Ag *K*α data are better when absorption is significant. Empirical corrections intended to correct for absorption also correct well for the effects of the highly focused IµS beams.

## 1. Introduction   

Microfocus sealed-tube X-ray sources have become standard in many laboratories because of their very low power consumption and minimal maintenance requirements (Coles & Gale, 2012 ▶; Schulz *et al.*, 2009 ▶). Cu *K*α and Mo *K*α microsources are already widely used, but the more recent commercial availability of silver anode microsources raises the question as to when Ag *K*α is preferable. The shorter wavelength enables a higher resolution to be achieved and results in a compressed diffraction pattern, which is particularly advantageous when the diffraction geometry is restricted, for example by a high-pressure cell (Saouane *et al.*, 2013 ▶). The strength of the absorption correlates with the wavelength of the incident beam: a short wavelength is generally less prone to absorption unless it is close to an absorption edge (Hamilton, 1965 ▶; Becker & Coppens, 1974*a* ▶,*b* ▶). In the case of large, strongly absorbing crystals, it is possible that reduced absorption with the silver anode could more than compensate for the decrease in the absolute scattering power of the crystal (which is proportional to λ^3^). The question of the optimal crystal size has been investigated for weakly absorbing crystals by Görbitz (1999 ▶), who used a system with a sealed tube source and monochromator.

The curved mirror optics used by both Mo and Ag microsources deliver a narrow beam with a slightly anisotropic profile, making accurate sample alignment essential (Arndt, 1990 ▶; Coles & Hursthouse, 2004 ▶; Storm *et al.*, 2004 ▶). The focal spot size of the beam is 110 and 90 µm for Mo *K*α and Ag *K*α, respectively (Hasse *et al.*, 2010 ▶). This highly focused beam makes a uniform homogeneous sample illumination impossible even for small crystals. In this paper, molybdenum and silver microsource data are compared for a variety of crystals with significant absorption in typical data collection situations. Although in these tests independent atom model (IAM) refinements were employed, the conclusions should also apply to data collected for charge density studies.

The *SADABS* program (Bruker, 2014 ▶) assumes that the corrected intensity is given by the product of an incident beam scale factor *S*(*n*), where *n* is the frame number, a diffracted beam factor *P*(*u*, *v*, *w*), where *u*, *v* and *w* are the direction cosines of the diffracted beam relative to crystal-fixed axes, and a spherical crystal factor *Q*(μ*r*, 2θ), where μ is the linear absorption coefficient and *r* the effective radius of the crystal: 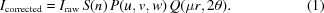
Similar approximations were used by Kopfmann & Huber (1968 ▶), North *et al.* (1968 ▶) and Huber & Kopfmann (1969 ▶) and in many subsequent papers and programs. There is one incident beam scale factor *S*(*n*) for each frame *n*, but in *SADABS* the values are interpolated according to the calculated rotation angle of the reflection relative to the rotation angles of the beginning and end of the frame. In addition, a restraint is applied that adjacent frames should have similar scale factors; this is essential when there are few (perhaps even zero) reflections that have their centres on a particular frame. The incident beam factor *S*(*n*) in *SADABS* corrects for crystal decomposition, intensity variations of the X-ray source, changes in the effective volume irradiated (possibly caused by the crystal not being accurately centred), beam inhomogeneity, and absorption by the crystal and its support. The plot of *S*(*n*) against the frame number *n* is a useful diagnostic (see below). The diffracted beam factor *P*(*u*, *v*, *w*) is based on spherical harmonics. Blessing (1995 ▶) also used spherical harmonics but applied them to both the incident and diffracted beams.

The empirical or multiscan correction involves refining the incident beam scale factors and spherical harmonic coefficients so that the intensities of equivalent reflections become more equal (Kopfmann & Huber, 1968 ▶; North *et al.*, 1968 ▶; Blessing, 1995 ▶). This is critically dependent on there being a high multiplicity of observations involving different paths through the crystal, so in general multiple scans about different rotation axes relative to the crystal are required. In *SADABS* the incident beam scale factors and spherical harmonic coefficients are refined in alternate half-cycles, so that each of these full-matrix refinements is linear. This has the advantage that no starting values are required and that each half-cycle converges in one iteration. After each half-cycle the weighted mean intensity of each reflection is calculated using robust/resilient weights as described by Blessing (1997 ▶), and the resulting weighted mean intensities are used as observations for fitting the least-squares parameters. Several double cycles are required, but the method is robust and fast. The spherical crystal term *Q*(μ*r*, 2θ) (Blessing, 1995 ▶) is applied only after the other parameters have been refined to convergence, because it has no effect on the agreement of the equivalent reflections. Since the spherical absorption factor *Q*(μ*r*, 2θ) is largest at low 2θ and decreases monotonically as 2θ increases, the effect of neglecting this term would be to cause the atomic displacement parameters to become too small or even negative (Katayama, 1986 ▶). If the crystals faces have been indexed and their distances from a reference point in the crystal determined, a numerical absorption correction based on Gaussian integration (Busing & Levy, 1957 ▶) may be performed in *SADABS* before the refinement of the other parameters. In such a case, lower-order spherical harmonics can be used in *P*(*u*, *v*, *w*). For X-ray beams from a sealed tube source that have been shaped by slits but not focused, this procedure works well because the assumption that the crystal is completely bathed in a uniform (top-hat profile) beam is valid, and it is even possible to use it to refine the linear absorption coefficient μ. As will be shown, this approach fails for the highly focused microsource beams.

After the determination of the scaling parameters, *SADABS* rejects severe outliers and scales the estimated standard deviations of the intensities so that they correspond statistically to the degree of agreement between the corrected intensities of the equivalent reflections. The equation used to scale the reflection standard deviations involves two parameters, *K* and *g*, that are refined so that the weighted mean square deviation χ^2^ is as close as possible to unity over the full range of intensities. Since there is no resolution-dependent term in this error model, plots of χ^2^ against resolution are a particularly effective diagnostic test; in an ideal case χ^2^ should be close to unity over the full ranges of intensity and resolution. In the work reported here, the current standard *SADABS* option of refining one overall *g* value and one *K* for each scan was adopted:
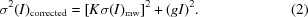



It should be noted that the current versions of *SADABS* and the programs *XDS* (Kabsch, 2010 ▶), *AIMLESS* (Evans & Murshudov, 2013 ▶) and *HKL-2000* (Borek *et al.*, 2003 ▶), which are very widely used for macromolecules, all use the same error model, an example of convergent evolution. This error model is justified by the fact that it results in values of χ^2^ that are close to unity throughout the full range of intensity and resolution, except sometimes for a small rise at very low resolution that is clearly indicative of a residual systematic error. This can be seen later in Fig. 5 (see §3.1) and for many thousands of data sets processed by *SADABS*. It is remarkable that this is achieved by the refinement of only two parameters, *K* and *g*. However recent versions of *SADABS* also allow these parameters to be held fixed (*e.g.* at 1 and 0, respectively), refined as overall values for all scans or refined separately for each scan. Here we have adopted the default *SADABS* option of refining separate *K* values for each scan (because they may be influenced by different scan speeds *etc*.) but only one overall *g* value. This error model has been criticized by Henn & Meindl (2010 ▶) and Jørgensen *et al.* (2012 ▶), who, however, do not explain why they prefer to ignore the standard statistical criterion that χ^2^ should be close to unity. A direct consequence of this error model is the characteristic shape of the Diederichs plot (Diederichs, 2010 ▶), a scatter plot of *I*/σ against log(*I*) for the unmerged data to assess the influence of systematic errors, shown later in Fig. 6 (§3.1), which has a limiting maximum value of *I*/σ given by 1/*g*.

## 2. Experimental   

### 2.1. Test crystals   

**Figure 1:**
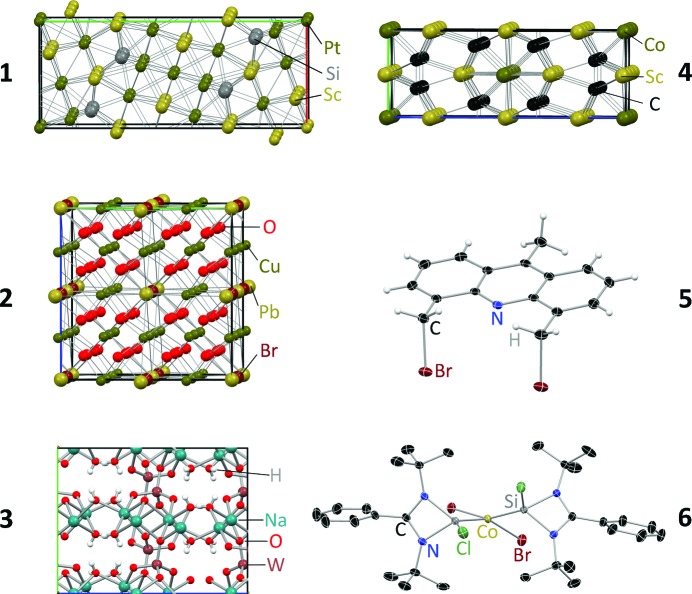
Test crystals.

Scandium platinate, **1** (Harmening *et al.*, 2010 ▶), murdochite, **2** (Dubler *et al.*, 1983 ▶), sodium tungstate, **3** (Farrugia, 2007 ▶), and scandium cobalt carbide, **4** (Rohrmoser *et al.*, 2007 ▶; Scherer *et al.*, 2010 ▶; Eickerling *et al.*, 2013 ▶), were used to represent inorganic compounds and minerals with medium to high absorption coefficients. Small crystals were chosen for this investigation in order to match the highly focused beams of the two microsources. Less strongly absorbing test crystals included a dibromoacridine derivative, **5** (Visscher, unpublished), and an inorganic cobalt complex, **6** (Azhakar *et al.*, 2013 ▶). See Fig. 1 ▶ and Table 1 ▶ for detailed information on each sample.

### 2.2. Diffractometer setup and data acquisition   

**Figure 2:**
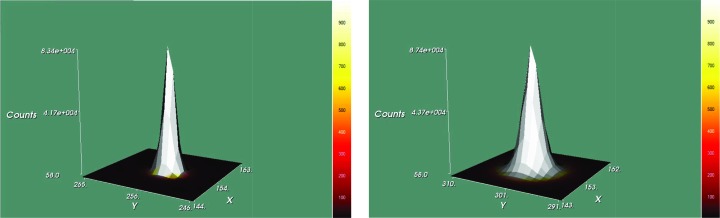
Reflection profiles as recorded by the scintillation phosphor for Mo *K*α (left) and Ag *K*α (right). 4 × 4 binning mode was used for both sources.

All experiments were performed on Bruker SMART APEX II systems based on D8 three-circle goniometers with Incoatec microfocus X-ray sources (IµS) and Incoatec QUAZAR mirror optics (Schulz *et al.*, 2009 ▶). The data were collected at 100 K crystal temperature (Mo source: Bruker CRYOFLEX; Ag source: Oxford Cryosystems CRYOSTREAM 700), 50 kV and 600 µA for both machines with an appropriate 0.5° ω scan strategy for the wavelength in question. Since no radiation damage to the crystals was expected, the same crystals were used to collect data successively on both diffractometers. Differences in scattering power and resolution for the two wavelengths led to differences in the data collection strategy and in the exposure times. Both diffractometers are equipped with Bruker APEX II area detectors that use Fairchild CCD6161 sensors. The only difference is the thickness of the scintillation phosphor, which results in a characteristic quantum yield of 160 e per X-ray photon for Mo *K*α and 204 e per X-ray photon for Ag *K*α. The detector on the Ag source uses a slightly thicker scintillation phosphor in order to compensate for the smaller gain caused by the shorter wavelength. A thicker scintillation phosphor increases the sensitivity but also increases the point spread function, which significantly broadens the reflection profiles (Gruner *et al.*, 2002 ▶), as can be seen in Fig. 2 ▶.

### 2.3. Data processing   

Data reduction was performed with *SAINT* (version 7.68A; Bruker, 2009 ▶) from the program package *APEX2* (version 2.2012.2-0; Bruker, 2009 ▶). The *SAINT* data reduction program uses either a predetermined or an internally derived and refined box size for the integration steps. The dimensions of this box are expected to be primarily determined by the mosaicity of the crystal, the point spread function of the detector and, where applicable, the *K*α_1_/*K*α_2_ splitting. As the same crystals were used with both sources, no changes in mosaicity were expected. However, in order to minimize systematic errors due to imprecise or improperly determined box sizes, the box size was always determined and refined by *SAINT* using a standard procedure. Data were collected up to a maximum resolution (max.) that was limited either by the scattering power of the sample or by the 2θ limit of the experimental setup. These limits are roughly 0.43 and 0.31 Å for the Mo and Ag sources, respectively, and are solely due to the different wavelengths since both sources were mounted on identical goniometers. The data for each crystal were then integrated to different resolution shells (1.00, 0.83, 0.79, 0.60, 0.43 and max. Å). This was done to facilitate the detection of resolution-dependent differences.

### 2.4. Scaling and ‘absorption’ corrections   

*SADABS* (version 2014/4) was employed for the incident beam scaling, determination of the spherical harmonic coefficients, outlier rejection and determination of the error model parameters. Additional tests were required to see if the empirical absorption correction method was a suitable treatment for the highly absorbing crystals, since the numerical correction requires well defined crystal faces. It was almost impossible to index the faces of the tiny crystals of **1** and **4** reliably, so the numerical and empirical absorption corrections were compared for the crystals of **2**, **3** and **5**, since these were larger than the width of the beam and had high linear absorption coefficients μ. It was anticipated that the numerical absorption correction would provide the best correction and that for the empirical correction it might be difficult to estimate the effective radius *r* for the additional spherical crystal correction. The validation of this correction involved a stepwise increase of the μ*r* value, followed by a comparison of the principal mean square atomic displacements of selected atoms with the values obtained by the numerical method. Satisfactory results were achieved when *r* was chosen so that it is biased towards the smallest crystal dimension; *e.g.* for a crystal with dimensions 0.1 × 0.2 × 0.3 mm and μ = 10 mm^−1^, 0.07 mm would be a good value for *r*, giving 0.7 for μ*r*.

### 2.5. Structure refinement   

All the structures were solved by either Patterson or direct methods with *SHELXS* (Sheldrick, 2008 ▶). They were refined by full-matrix least squares against *F* ^2^ using *SHELXL-2014/3* with the help of the *SHELXle* graphical user interface (Hübschle *et al.*, 2011 ▶). All non-H atoms were refined with anisotropic displacement parameters (ADPs). The H atoms were set to idealized positions and refined using a riding model with their isotropic displacement parameters constrained to be 1.5 times the equivalent isotropic displacements of the atoms to which they were attached for methyl H atoms and 1.2 times for all other H atoms. The bromine/chlorine disorder in **2** was treated with EADP/EXYZ constraints in *SHELXL-2014/3*. In compound **6** the chlorine/bromine disorder and the rotational disorder of the tertiary butyl group attached to N1 were refined using distance and ADP restraints.

## 3. Results   

### 3.1. Quality of the processed data   

Table 2 ▶ shows the quality indicators after scaling and correction. For this table the data were truncated to the highest common resolution, but if the crystal diffracted further with Ag *K*α than could be achieved with Mo *K*α and the experimental geometry employed, these Ag *K*α data are also reported. The data collection strategies were optimized for the wavelength in question, which resulted in only slightly longer total data collection times for Ag *K*α. To some extent, the larger number of reflections recorded per frame for Ag *K*α and the corresponding reduction in the number of different detector 2θ settings required compensates for the higher Mo *K*α flux. To reduce the influence of the multiplicity on the quality indicators, the multiplicity-independent *R*_r.i.m._ and *R*_p.i.m._ (Weiss, 2001 ▶) are shown. Except for sample **6**, which gave the weakest diffraction and would probably have benefited from a longer total data collection time with Ag *K*α radiation, these *R* values and 〈*I*/σ〉 for the merged data are very comparable for the two sources for data to the same resolution. The broader reflection profile for Ag *K*α (Fig. 2 ▶) requires the use of slightly larger integration boxes and hence involves a larger contribution from the background noise. However, this appears to have had little influence on the data from these relatively strongly diffracting crystals.

Table 2 ▶ also shows the asymptotic limiting value of *I*/σ for infinite intensity (calculated by *SADABS* as 1/*g* from its error model) and the average number of reflections collected per second. This is calculated by dividing the total time required for the data collection by the number of reflections measured, which in most of the cases is higher for the Ag *K*α data.

**Figure 3:**
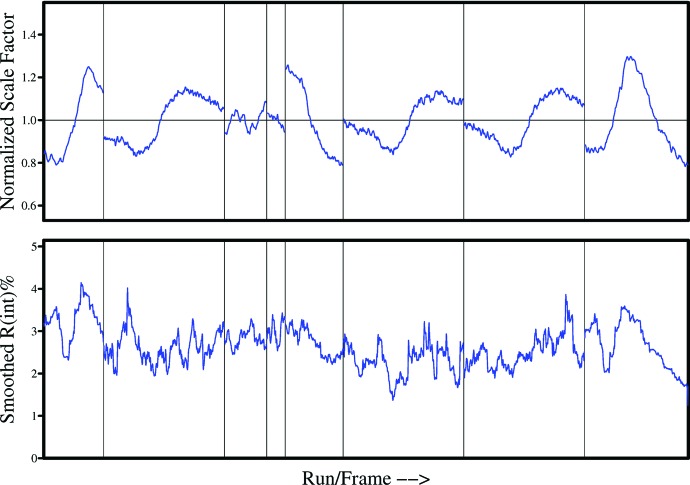
Incident beam scale factor *S*(*n*) and merging *R*_int_ as output by *SADABS* for the strongly absorbing crystal **2** with Ag *K*α radiation. It should be noted that the smoothing algorithm for the *R*_int_ plots was changed in *SADABS 2014/4* to make these plots more informative.

**Figure 4:**
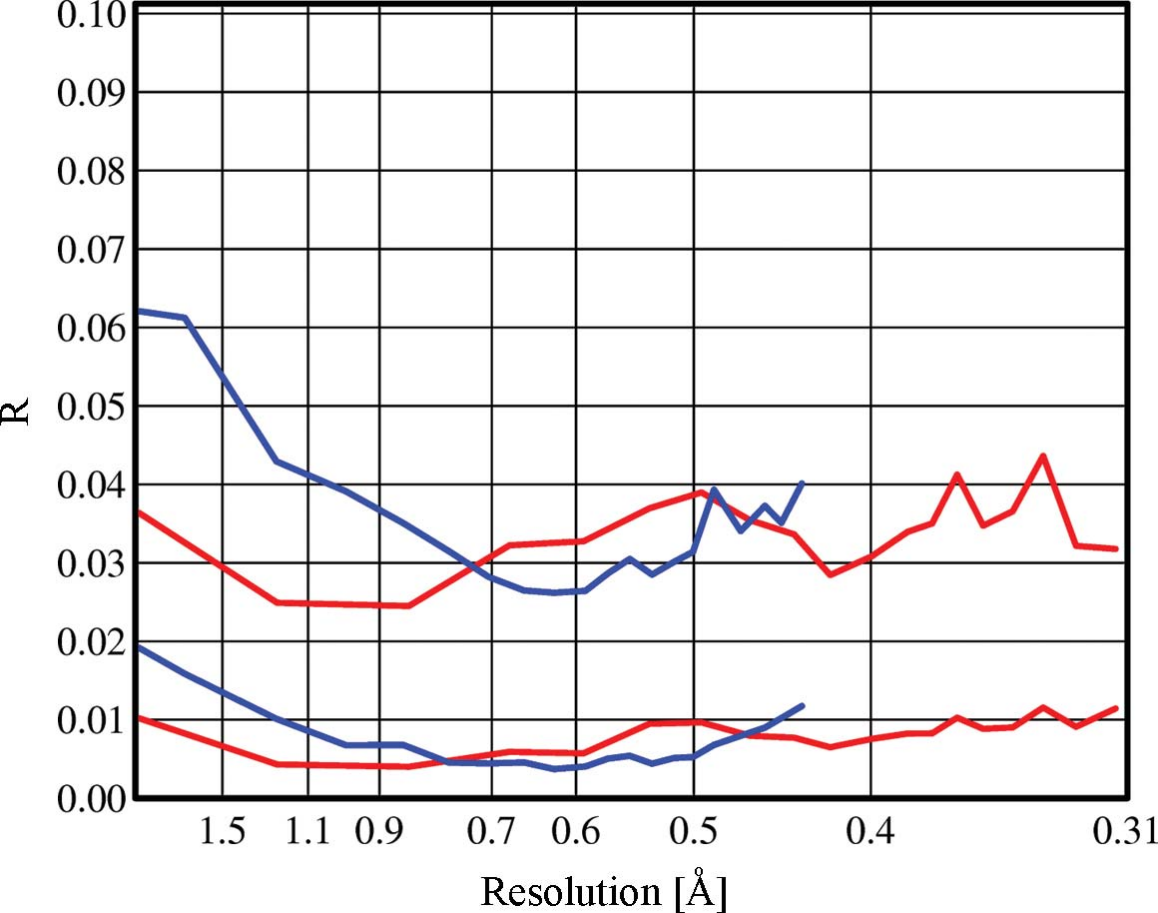
*R*_r.i.m._ (upper curves) and *R*_p.i.m._ (lower) after correction as a function of the resolution in ångström for Ag (red) and Mo (blue) for the strongly absorbing crystal **2**. This figure was prepared with the *XPREP* (Bruker, 2014 ▶) program.

**Figure 5:**
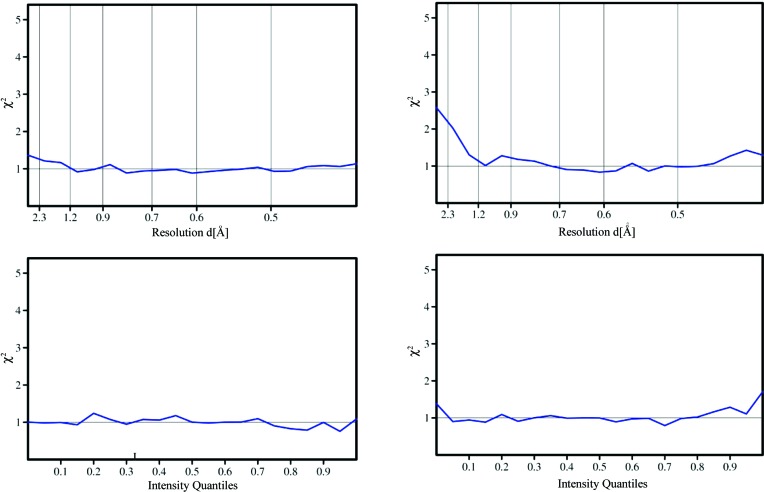
χ^2^ after applying corrections and deriving the error model for crystal **2**. Ag (left) and Mo (right) radiation. χ^2^ = mean{*N*∑(*I* − 〈*I*〉)^2^/(*N* − 1)∑[s.u.^2^(*I*)]} (*N* equivalents).

As shown in Fig. 3 ▶, the variations in the incident beam correction factor *S*(*n*) can be substantial, even for Ag *K*α radiation. Despite this, the *R*_r.i.m._ and *R*_p.i.m._ values after correction (red lines in Fig. 4 ▶) are low and show little systematic variation with resolution. The corresponding values for Mo *K*α (blue lines in Fig. 4 ▶) are similar at higher resolution but increase significantly at low resolutions, indicating that the empirical absorption correction is less effective at correcting for the even higher absorption with molybdenum radiation. The χ^2^ plots for the same experiments in Fig. 5 ▶ again show a more pronounced rise at low resolutions for the molybdenum data; however, these plots also demonstrate that the corrections have been very effective for both sources, even for this highly absorbing sample. Since the error model has not been fitted as a function of the resolution, a flat curve close to a χ^2^ of unity for the full resolution range is a particularly good validation of the quality of the corrected data. Convincing χ^2^ plots were obtained in all the analyses reported here (see supporting information^1^).

**Figure 6:**
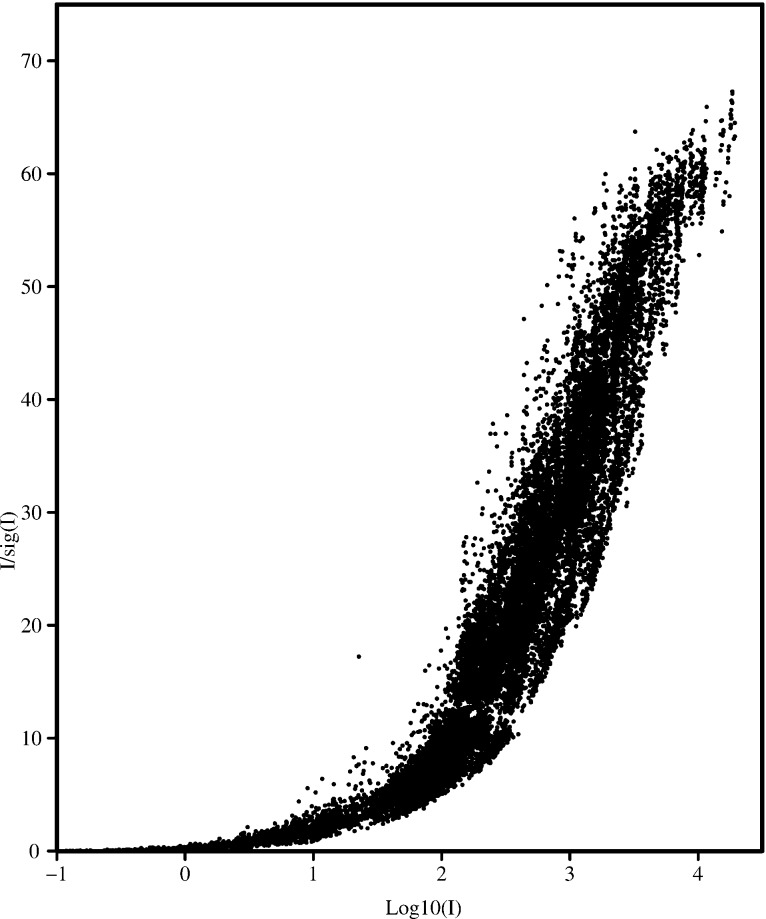
Diederichs plot of *I*/σ against log(*I*) for sample **4** for Ag *K*α data to 0.43 Å.

Fig. 6 ▶ shows the Diederichs plot prepared using *SADABS* for the Ag *K*α data to 0.43 Å resolution for sample **4**. A limiting value greater than 30 for *I*/σ at infinite intensity is regarded as good for synchrotron data and is taken to indicate that the data are relatively free from systematic errors. With the exception of the highly absorbing sample **1**, the values reported here are all higher than 30.

The limiting *I*/σ values for the unmerged data are relatively constant for the same sample and do not vary much with the resolution threshold, supporting the idea that this is a robust indication of the extent of systematic error for a given crystal and experimental arrangement. On the other hand, the mean 〈*I*/σ〉 values for the merged data are clearly correlated with the multiplicity, which tends to decrease at the highest resolution. For the strongly absorbing sample **2**, the merging *R* values are lower for the Ag *K*α data, but the opposite is true for the less strongly absorbing sample **6**. Overall the precision of the Ag *K*α and Mo *K*α data is comparable.

### 3.2. Comparison of model quality   

After the full structure refinement, the *R*1 value calculated using all data, the *wR*2 value (minimized in the full-matrix least-squares refinement) and the residual electron density Δρ were compared at both the maximum resolution achieved and the standard resolution of 0.83 Å. Δρ was calculated as the difference between the highest and lowest residual density in a weighted difference Fourier map.

For crystals **2**, **3** and **5** it proved possible to index the crystal faces and compare the numerical and empirical absorption corrections. However the attempts to refine the absorption coefficient μ, although this works well for conventional sealed tube sources without focusing optics, were not satisfactory. Especially for the Ag *K*α data, μ refined to unreasonably small values or even to zero. This problem may be attributed to the use of highly focused beams, the Ag *K*α source having the most highly focused beam. When the numerical correction is combined with lower-order spherical harmonics (even/odd orders 4/1), the merging *R* values and the *R*1 values for the *SHELXL* refinement (shown in Table 3 ▶) were extremely similar to those obtained using no numerical correction but higher-order spherical harmonics (specified in Table 1 ▶) plus a spherical crystal correction *Q*(μ*r*, 2θ). In both cases the incident beam term *S*(*n*) is responsible for about half the correction. It is thus debatable whether the numerical correction is justified. In practice an effective crystal radius *r* for the spherical correction *Q*(μ*r*, 2θ) biased towards half the smallest crystal diameter gives an adequate spherical crystal correction.

For ten of the 12 combinations of crystal and resolution cutoff shown in Table 3 ▶, both *R*1 and *wR*2 were lower for the Ag *K*α data. The residual density values show a similar trend but are not quite as decisive. The *R*1 and *wR*2 values are significantly lower for Ag (average values *R*1 0.0178, *wR*2 0.0398) than for Mo (*R*1 0.0197, *wR*2 0.0485). Thus, although the data precision (Table 2 ▶) is comparable for the two sources, the Ag data are clearly more accurate (Table 3 ▶). These low *R* factors (three of the *R*1 values for all data are below 1%) confirm that the empirical corrections have performed remarkably well, despite the unfavourable combination of highly focused beams and relatively high absorption.

For the refinement of structures **2** and **4** against data truncated to the standard (*Acta Crystallographica*) requirement of 0.83 Å, the data-to-parameter ratios are low (5.27 and 6.33, respectively). Since the scattering is dominated by the Pb and Br atoms in the case of **2**, the O atoms cannot reliably be refined. However, with data to 0.43 Å the data-to-parameter ratio is 28.55 and there are no problems refining the O atoms. It should be standard practice to collect data to the highest possible resolution when both heavy and light atoms are present.

## 4. Conclusions   

The empirical correction employed in *SADABS* performed remarkably well for strongly absorbing crystals despite the highly focused microsource beams, leading to very low *R* factors for the refined structures. While the precision of the corrected intensities was comparable for both Ag *K*α and Mo *K*α microsources, their accuracy was higher for the silver source because of the reduced absorption. For strongly absorbing crystals the Ag *K*α microsource data were in general less affected by systematic errors than the Mo *K*α data. The application of a numerical absorption correction did not improve the results. Clearly, the assumption that the crystal is completely bathed in a uniform X-ray beam is not valid for highly focused X-ray optics. However, when the empirical approach is used it is important to obtain a good estimate of the effective crystal radius for the correction term *Q*(μ*r*, 2θ). An estimate of *r* biased towards half the smallest crystal diameter is an adequate approximation.

## Supplementary Material

Crystal structure: contains datablock(s) global, 1-(Ag), 1-(Mo), 2-(Ag), 2-(Mo), 3-(Ag), 3-(Mo), 4-(Ag), 4-(Mo), 5-(Ag), 5-(Mo), 6-(Ag), 6-(Mo). DOI: 10.1107/S1600576714022985/aj5242sup1.cif

Structure factors: contains datablock(s) 1-(Ag). DOI: 10.1107/S1600576714022985/aj52421-Agsup2.hkl

Structure factors: contains datablock(s) 1-(Mo). DOI: 10.1107/S1600576714022985/aj52421-Mosup3.hkl

Structure factors: contains datablock(s) 2-(Ag). DOI: 10.1107/S1600576714022985/aj52422-Agsup4.hkl

Structure factors: contains datablock(s) 2-(Mo). DOI: 10.1107/S1600576714022985/aj52422-Mosup5.hkl

Structure factors: contains datablock(s) 3-(Ag). DOI: 10.1107/S1600576714022985/aj52423-Agsup6.hkl

Structure factors: contains datablock(s) 3-(Mo). DOI: 10.1107/S1600576714022985/aj52423-Mosup7.hkl

Structure factors: contains datablock(s) 4-(Ag). DOI: 10.1107/S1600576714022985/aj52424-Agsup8.hkl

Structure factors: contains datablock(s) 4-(Mo) Structure factors: contains datablock(s) 4-(Mo). DOI: 10.1107/S1600576714022985/aj52424-Mosup9.hkl

Structure factors: contains datablock(s) 4-(Mo) Structure factors: contains datablock(s) 4-(Mo). DOI: 10.1107/S1600576714022985/aj52424-Mosup9.hkl

Structure factors: contains datablock(s) 5-(Ag). DOI: 10.1107/S1600576714022985/aj52425-Agsup10.hkl

Structure factors: contains datablock(s) 6-(Ag). DOI: 10.1107/S1600576714022985/aj52426-Agsup12.hkl

Structure factors: contains datablock(s) 6-(Mo). DOI: 10.1107/S1600576714022985/aj52426-Mosup13.hkl

Supporting information file. DOI: 10.1107/S1600576714022985/aj5242sup14.pdf

CCDC references: 1029932, 1029933, 1029934, 1029935, 1029936, 1029937, 1029938, 1029939, 1029940, 1029941, 1029942, 1029943

## Figures and Tables

**Table 1:** Experimental setup and sample characteristics

	Space group	Crystal dimensions (mm)	*r* (mm)	Source	(mm^1^)	*r*	Spherical harmonics	Maximum resolution ()	Reflections: measured/unique
**1**	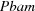	0.06/0.04/0.02	0.014	Ag	65.25	0.919	8/7	0.33	88135/6527
				Mo	121.02	1.705		0.43	51469/3183
**2**	*F* 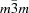	0.12/0.11/0.09	0.049	Ag	20.50	1.009	8/7	0.31	14474/770
				Mo	38.25	1.876		0.43	10420/313
**3**	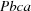	0.03/0.05/0.11	0.021	Ag	10.16	0.214	8/7	0.40	154303/10927
				Mo	18.84	0.397		0.44	84934/8314
**4**	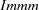	0.08/0.05/0.05	0.026	Ag	5.02	0.129	8/7	0.33	25448/1590
				Mo	9.78	0.251		0.43	11127/704
**5**	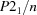	0.20/0.16/0.15	0.078	Ag	3.16	0.246	6/3	0.79	55453/3161
				Mo	5.90	0.459		0.79	35888/3130
**6**	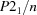	0.08/0.06/0.02	0.018	Ag	1.53	0.027	8/5	0.79	62614/8300
				Mo	2.87	0.051		0.79	96806/8338

**Table 2:** Data quality indicators

	Source	Resolution ()	Completeness (%)	Multiplicity	*I*/	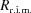	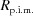	Exposure time	Unmerged *I*/ limit	Reflections per second
**1**	Ag	0.33	97.6	13.06	27.97	0.0718	0.0181	60120	16.4	0.18
		0.43	99.4	18.93	40.02	0.0619	0.0143		18.1	0.05
		0.83	99.8	12.88	45.42	0.0403	0.0113		21.3	0.04
	Mo	0.43	100	15.92	39.12	0.0491	0.0120	1590	17.0	0.02
		0.83	100	14.80	40.76	0.0464	0.0125		17.8	0.07
**2**	Ag	0.31	99.1	18.63	81.63	0.0298	0.0061	560	33.6	0.23
		0.43	100	25.27	112.20	0.0255	0.0051		40.1	0.17
		0.83	100	34.14	196.24	0.0214	0.0045		56.5	0.13
	Mo	0.43	100	33.19	125.77	0.0361	0.0071	560	29.1	0.09
		0.83	100	30.43	127.70	0.0370	0.0084		27.6	0.04
**3**	Ag	0.40	99.9	14.10	48.46	0.0301	0.0072	20120	43.9	1.06
		0.44	99.9	14.87	58.00	0.0282	0.0066		49.3	0.87
		0.83	100	25.96	105.23	0.0247	0.0049		44.8	0.01
	Mo	0.44	99.4	10.15	45.04	0.0316	0.0086	1060	37.1	0.49
		0.83	100	15.09	81.89	0.0241	0.0062		48.1	0.02
**4**	Ag	0.33	83.3	13.34	61.51	0.0274	0.0059	2060	54.9	0.11
		0.43	100	25.58	112.10	0.0247	0.0050		64.7	0.10
		0.83	100	21.39	194.77	0.0160	0.0038		82.5	0.16
	Mo	0.43	99.7	15.73	122.91	0.0208	0.0040	2060	66.1	0.04
		0.83	100	30.71	215.43	0.0216	0.0037		56.8	0.16
**5**	Ag	0.79	99.8	17.49	56.22	0.0323	0.0072	10	36.1	1.28
		0.83	100	18.35	60.84	0.0312	0.0068		36.4	0.92
	Mo	0.79	100	11.45	63.01	0.0242	0.0067	10	44.2	0.75
		0.83	100	11.92	67.22	0.0234	0.0064		44.0	0.74
**6**	Ag	0.79	99.5	7.51	29.94	0.0407	0.0145	3040	34.4	0.73
		0.83	99.5	7.71	33.01	0.0388	0.0136		38.0	0.68
	Mo	0.79	99.8	11.56	44.49	0.0290	0.0075	30	33.6	0.80
		0.83	99.8	12.43	49.87	0.0279	0.0070		34.4	0.75

**Table 3:** Selected quality criteria after structure refinement

	Resolution ()	Source	*R*1 (all data)	*wR*2	(e^3^)	Data/parameter	*R*1†
**1**	0.83	Ag	0.0133	0.0283	2.64	10.64	
		Mo	0.0216	0.0665	4.43	10.60	
	0.43	Ag	0.0219	0.0391	7.34	71.93	
		Mo	0.0262	0.0678	13.15	70.86	
**2**	0.83	Ag	0.0170	0.0566	1.68	5.27	0.0166
		Mo	0.0138	0.0360	1.33	5.27	0.0128
	0.43	Ag	0.0201	0.0469	9.69	28.55	0.0201
		Mo	0.0196	0.0451	7.07	28.45	0.0193
**3**	0.83	Ag	0.0080	0.0193	0.80	11.87	0.0081
		Mo	0.0094	0.0215	0.98	11.91	0.0092
	0.44	Ag	0.0151	0.0228	4.01	79.87	0.0151
		Mo	0.0172	0.0326	5.34	79.83	0.0165
**4**	0.83	Ag	0.0129	0.0354	0.77	6.33	
		Mo	0.0157	0.0408	1.21	6.33	
	0.43	Ag	0.0099	0.0254	1.25	39.11	
		Mo	0.0121	0.0327	1.70	39.11	
**5**	0.83	Ag	0.0193	0.0470	1.21	14.62	0.0193
		Mo	0.0197	0.0491	1.34	14.52	0.0198
	0.79	Ag	0.0206	0.0488	1.47	16.94	0.0205
		Mo	0.0211	0.0541	1.37	16.77	0.0211
**6**	0.83	Ag	0.0237	0.0488	0.54	16.00	
		Mo	0.0252	0.0572	0.63	16.07	
	0.79	Ag	0.0260	0.0506	0.66	18.55	
		Mo	0.0278	0.0593	0.65	18.64	

† *R*1 values for the refined structure after application of a numerical absorption correction based on the measured crystal faces and the absorption coefficient calculated from the known unit-cell contents. The other *R* values in this table were obtained using the empirical correction.

## Footnotes

1 Supporting information is available from the IUCr electronic archives (Reference: AJ5242).
