# Supplementary material for: Comparison of silver and molybdenum microfocus X-ray sources for single-crystal structure determination
Source: J Appl Crystallogr. 2015 Jan 30;48(Pt 1):3–10. doi: 10.1107/S1600576714022985 (PMC4453166; doi:10.1107/S1600576714022985)
Supplement: Supplementary file 13 [file j-48-00003-sup14.pdf]

JOURNAL OF APPLIED CRYSTALLOGRAPHY

**Volume 48 (2015)**

**Supporting information for article:**

# Comparison of silver and molybdenum microfocus X-ray sources for single-crystal structure determination

**Lennard Krause, Regine Herbst-Irmer, George M. Sheldrick and Dietmar Stalke**

## S1. *SADABS* Diagnostic plots

The following plots are generated from *SADABS* and show the variation of the normalized scale factor and smoothed $R_{int}$ as a function of the frame number. It should be noted that the smoothing algorithm for the R(int) plots was changed in *SADABS* 2014/4 to make these plots more informative.

**Data of sample 1:**

Ag

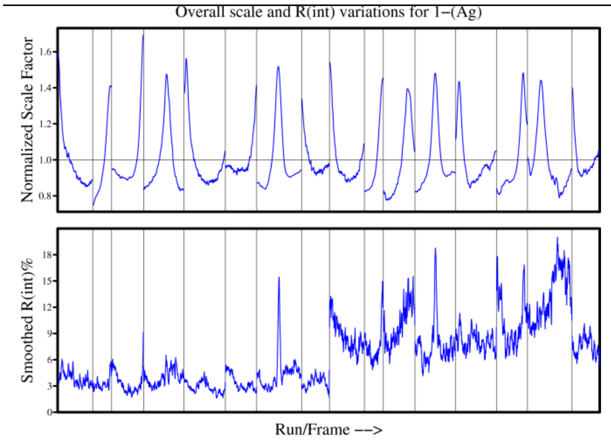


Mo

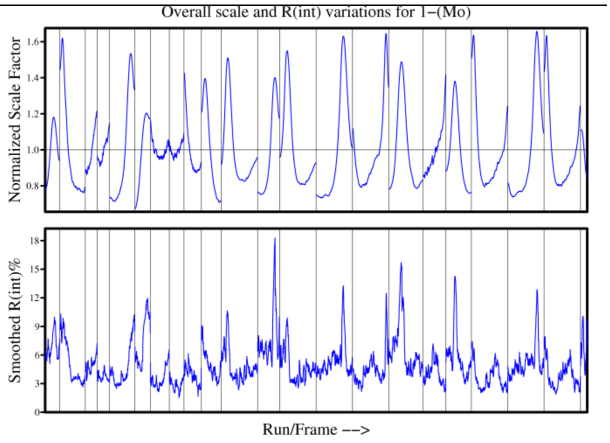


**Data of sample 2:**

Ag

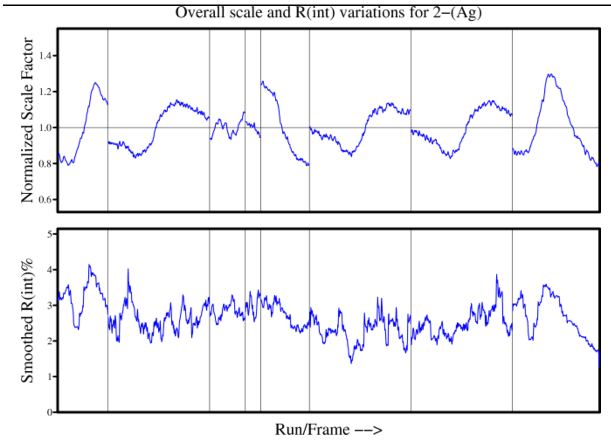


Mo

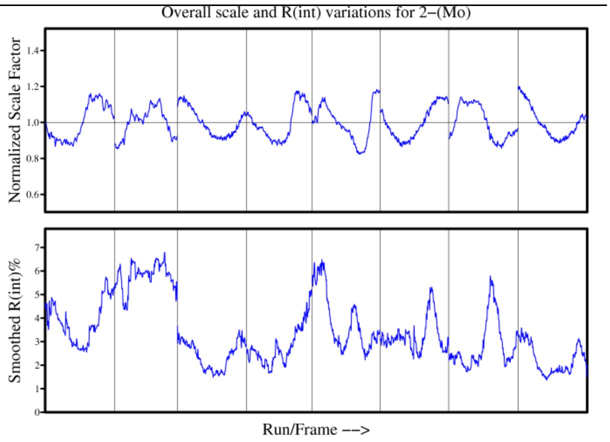

**Data of sample 3:**

Ag Mo

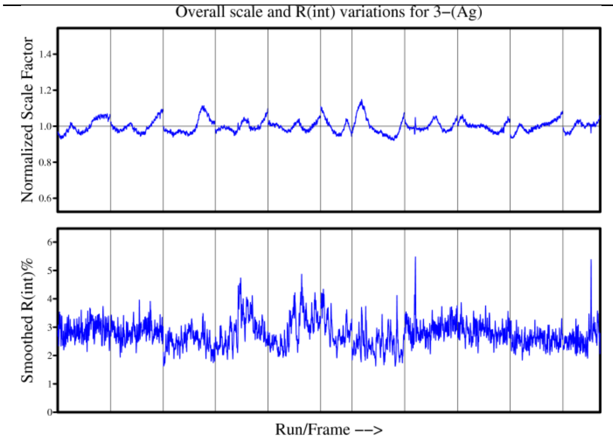


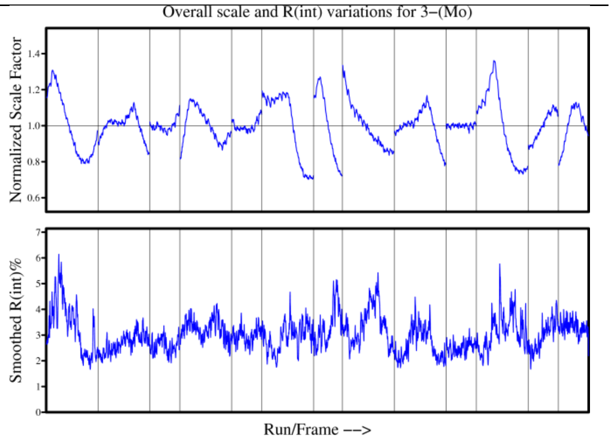


**Data of sample 4:**

Ag Mo

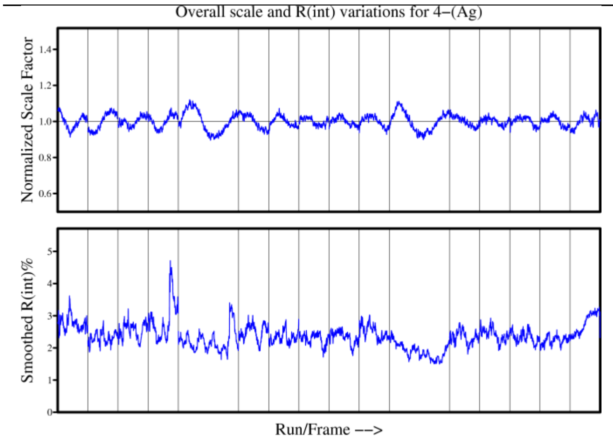


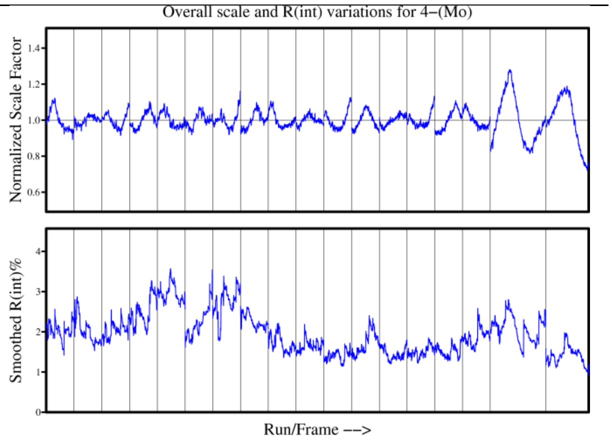

**Data of sample 5:**

Ag Mo

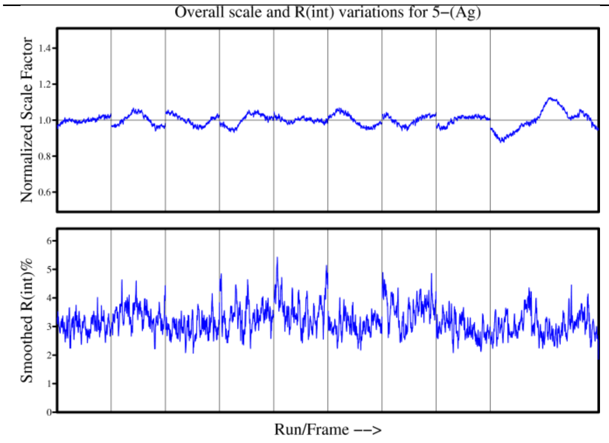


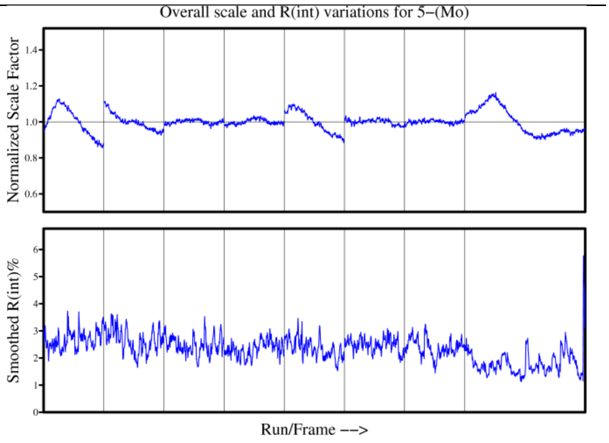


**Data of sample 6:**

Ag Mo

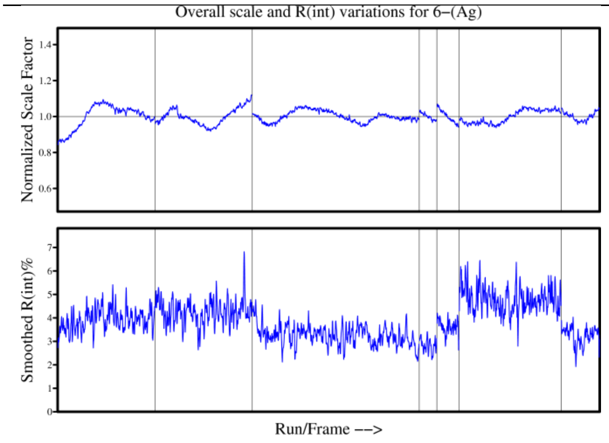


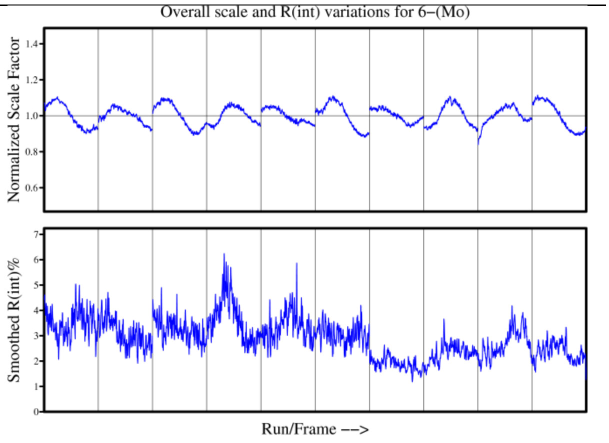

The following Plots are presented as an overlay of the two data sets, one corrected with the numerical (red) and the other with the empirical (blue) absorption correction.

**Data of sample 2:**

Ag

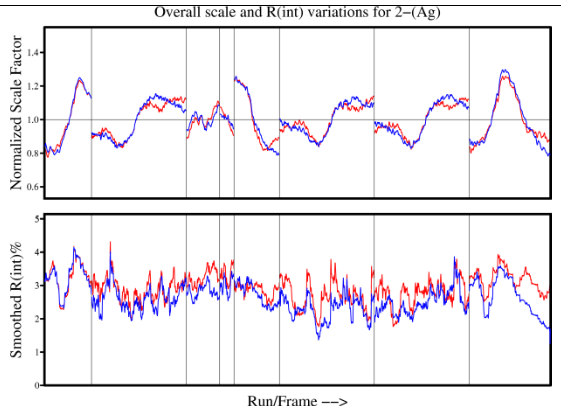


Mo

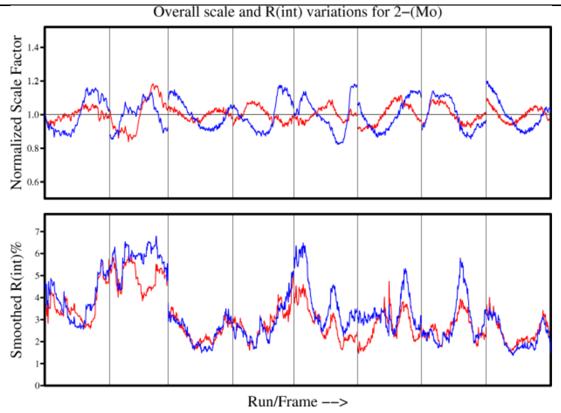

**Data of sample 3:**

Ag | Mo

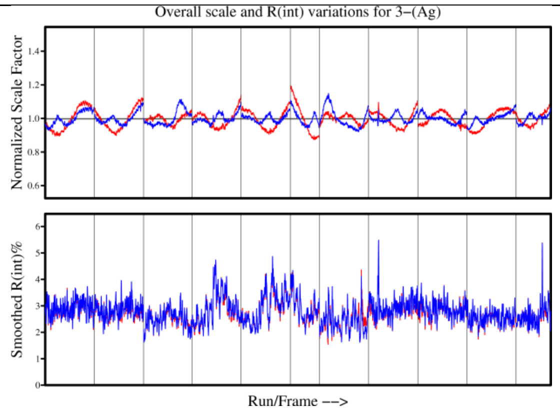


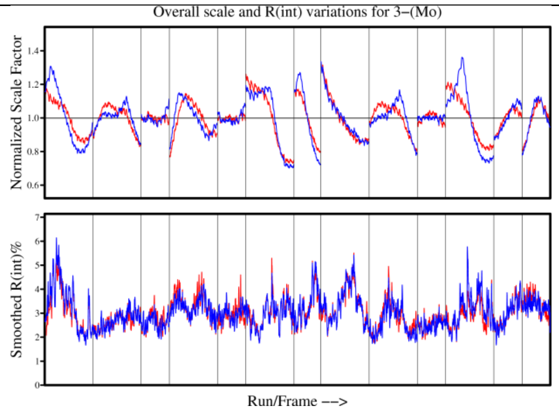


**Data of sample 5:**

Ag | Mo

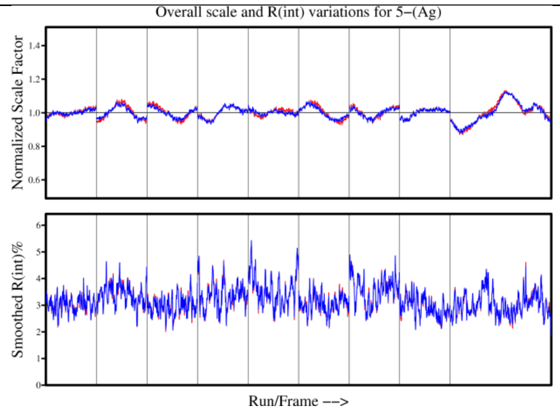


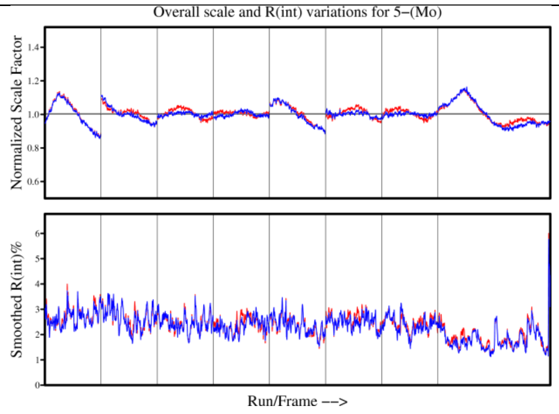

The following plots are generated from *SADABS* and show the variation of R(int), R(sigma) (upper) and |E²-1| (lower) as a function of resolution.

**Data of sample 1:**

Ag Mo

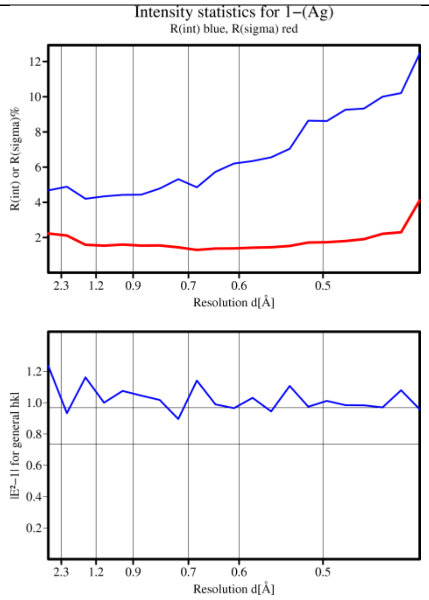


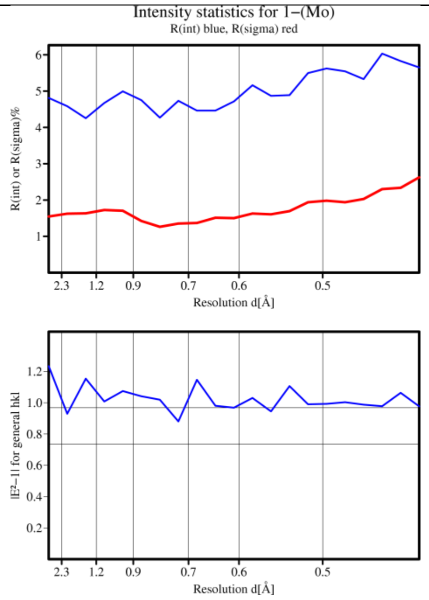


**Data of sample 2:**

Ag Mo

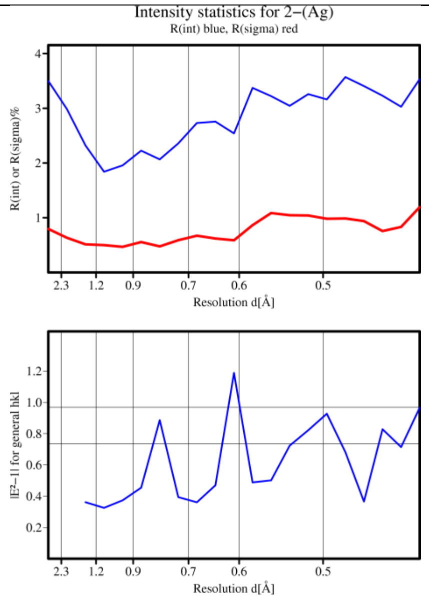


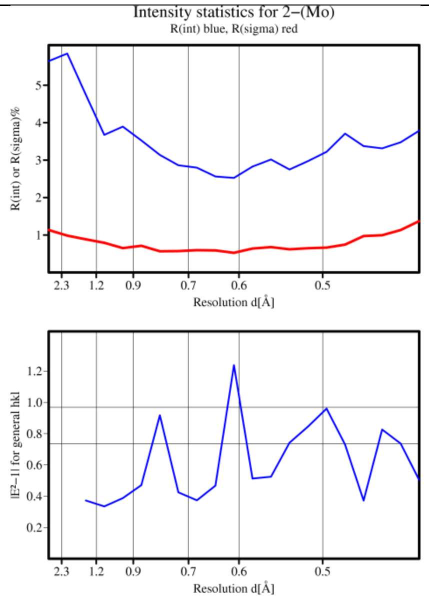

**Data of sample 3:**

Ag

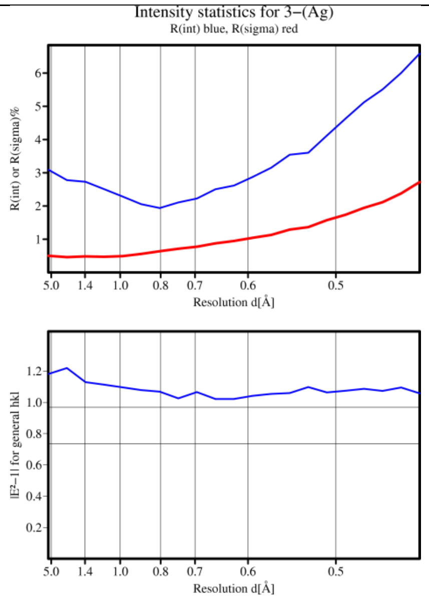


Mo

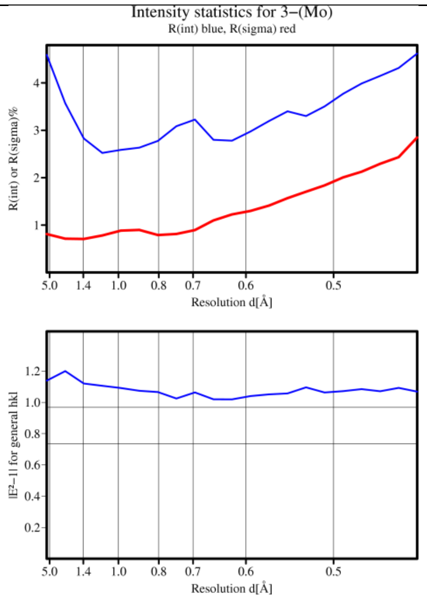


**Data of sample 4:**

Ag

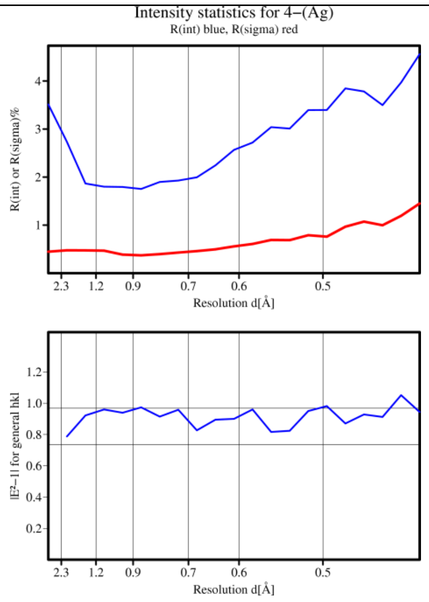


Mo

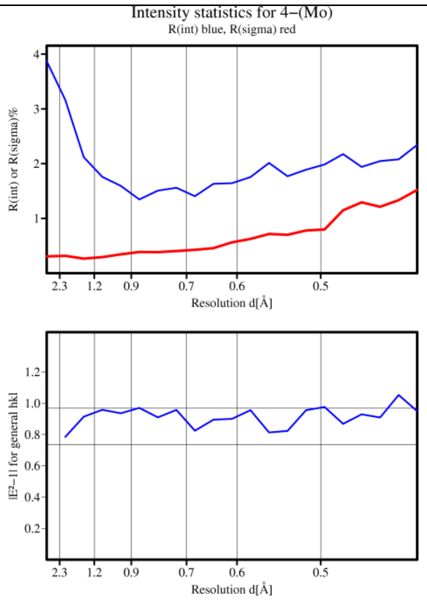

**Data of sample 5:**

Ag

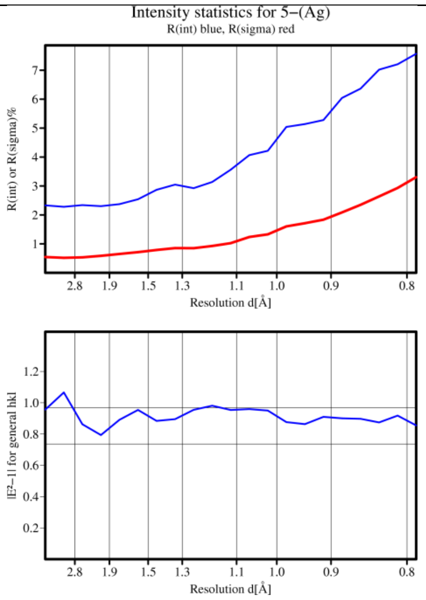


Mo

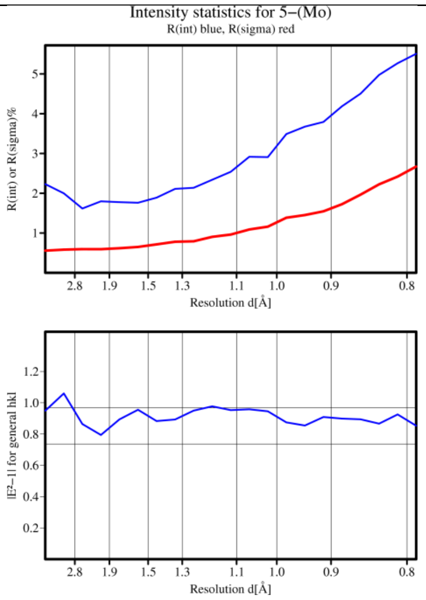


**Data of sample 6:**

Ag

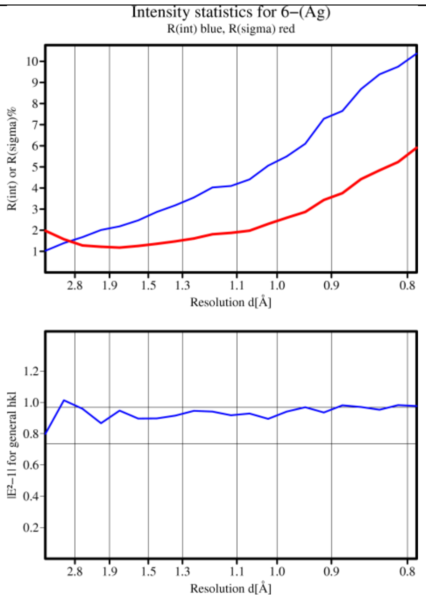


Mo

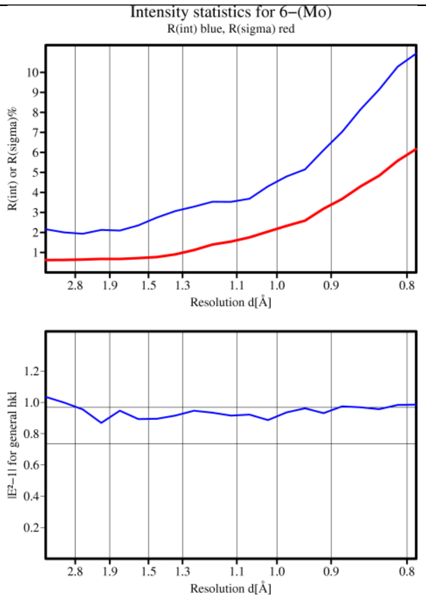

The following plots are generated from *SADABS* and show the variation of $\chi^2$ as a function of resolution and intensities.

**Data of sample 1:**

Ag

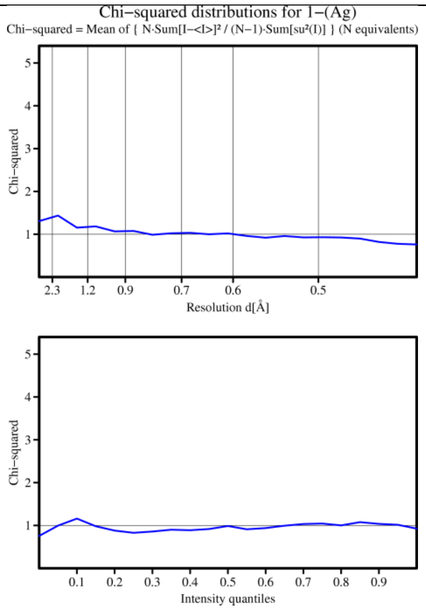


Mo

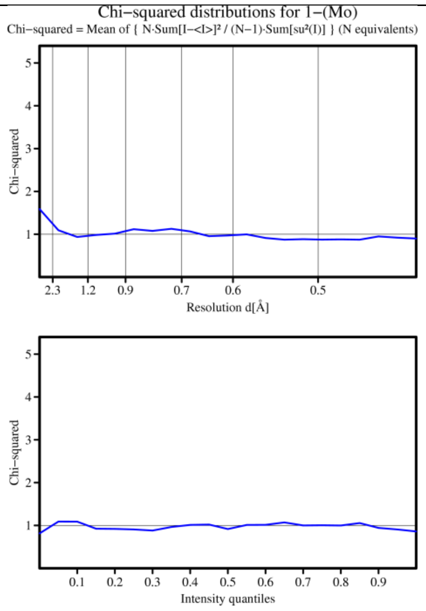


**Data of sample 2:**

Ag

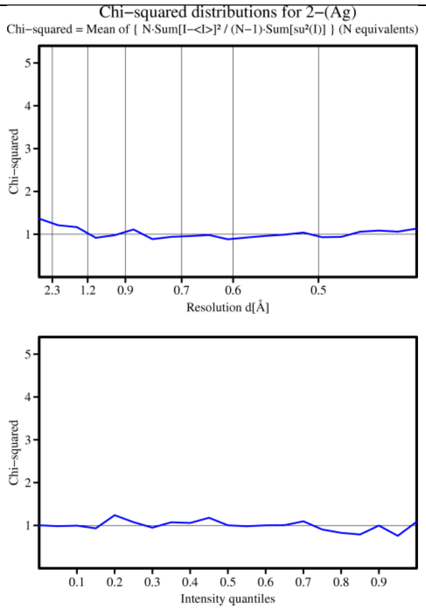


Mo

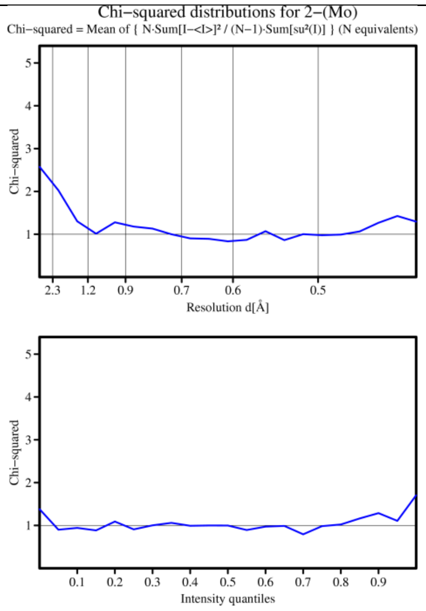

**Data of sample 3:**

Ag

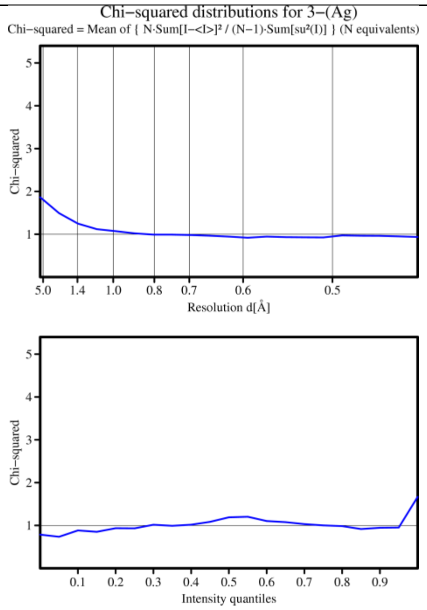


Mo

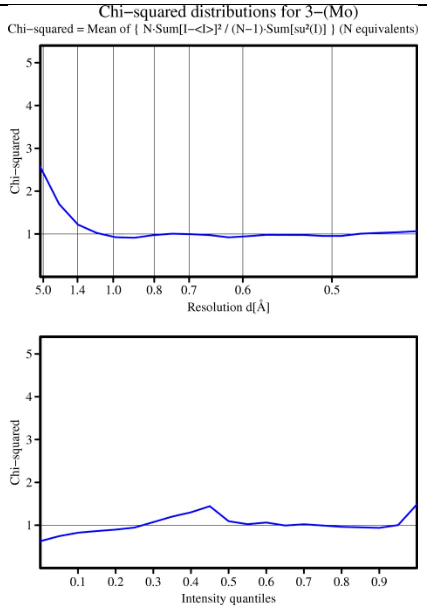


**Data of sample 4:**

Ag

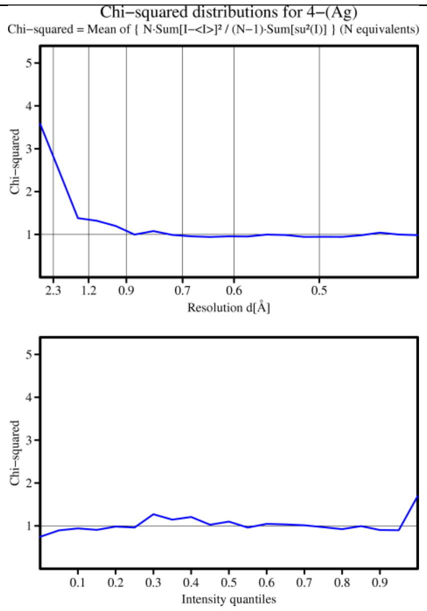


Mo

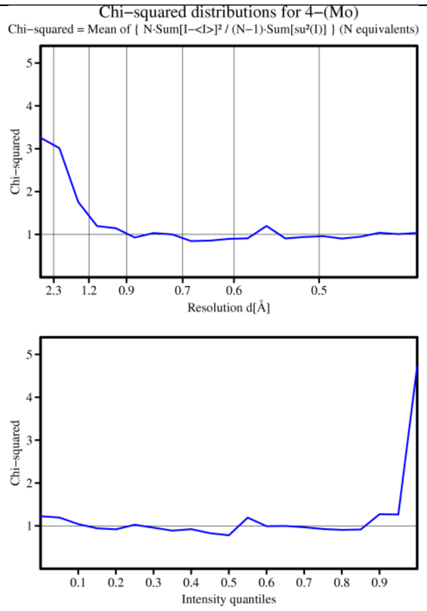

**Data of sample 5:**

Ag

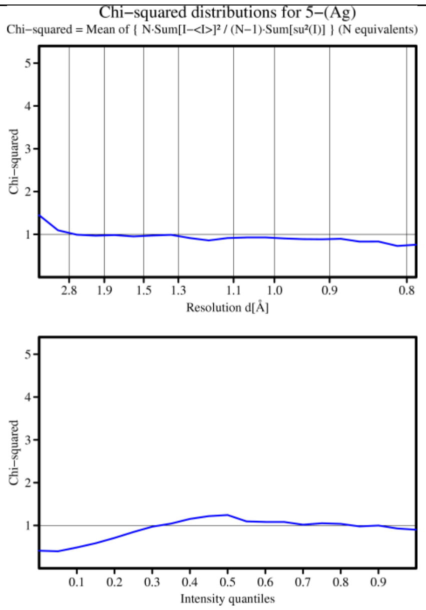


Mo

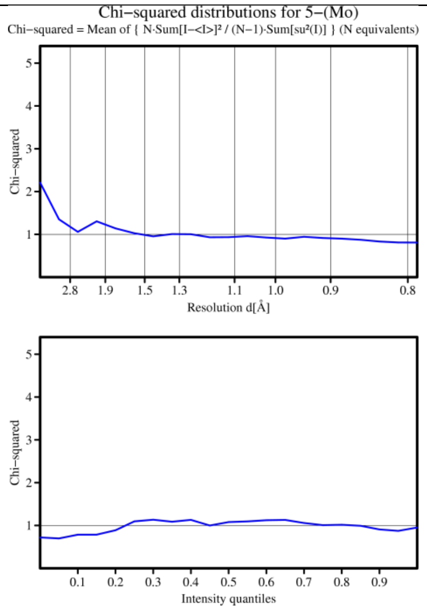


**Data of sample 6:**

Ag

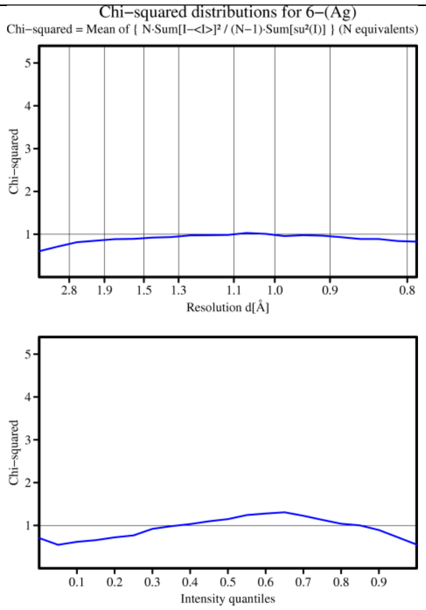


Mo

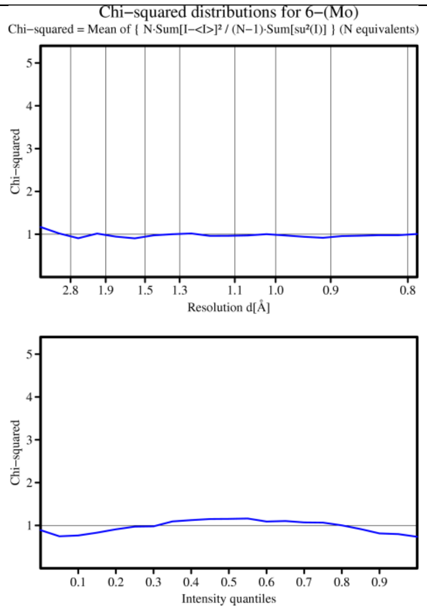

## S2. Significance of the data

In 2010, Diederichs suggested an indicator which is calculated after the data reduction to be used to estimate the systematic instrument error of the x-ray source. The value of this indicator is the highest [I/σ(I)] value the given experimental setup can produce.

**Data of sample 1:**

Ag

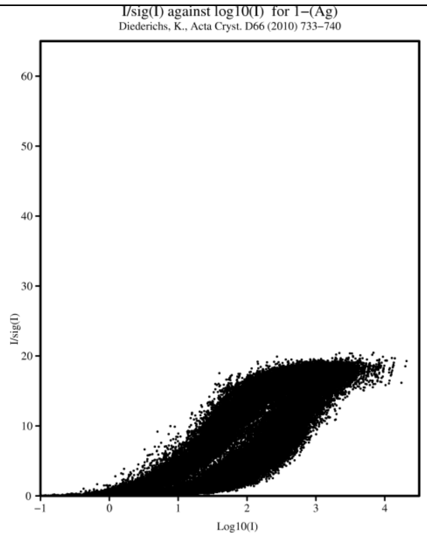


Mo

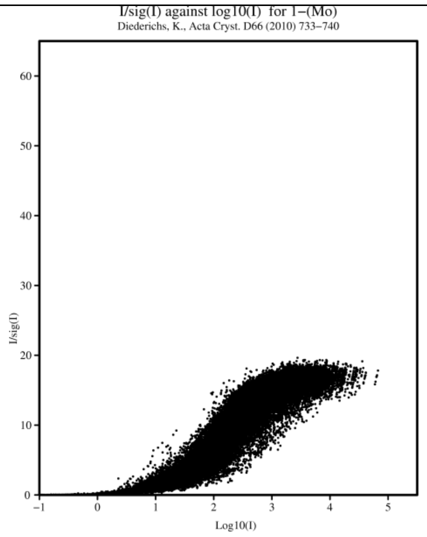


**Data of sample 2:**

Ag

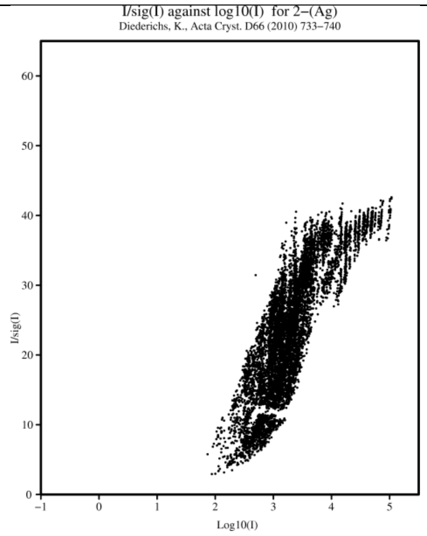


Mo

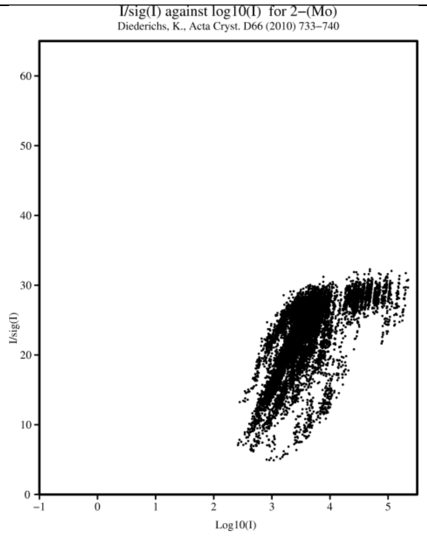

**Data of sample 3:**

| Ag | Mo |
|---|---|
| 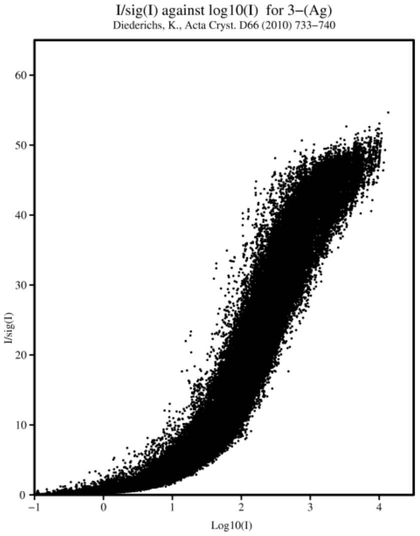 | 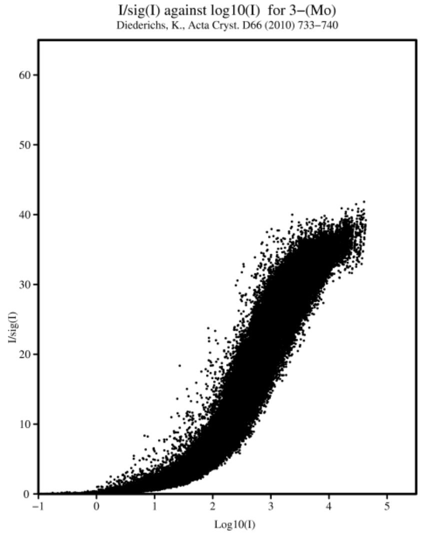 |

**Data of sample 4:**

| Ag | Mo |
|---|---|
| 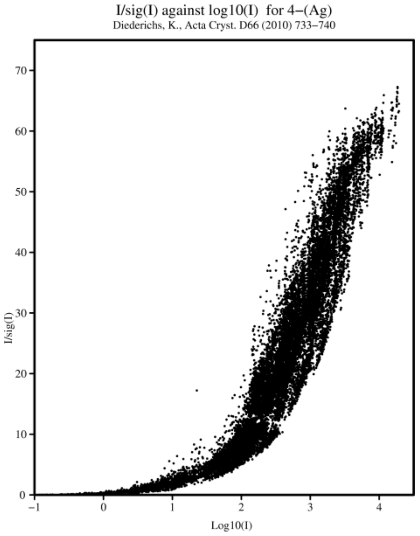 | 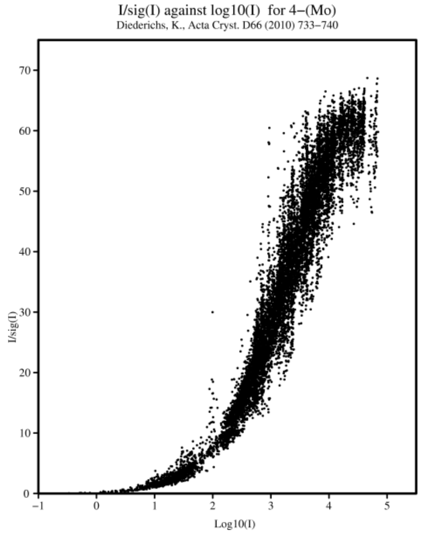 |

**Data of sample 5:**

| Ag | Mo |
|---|---|
| 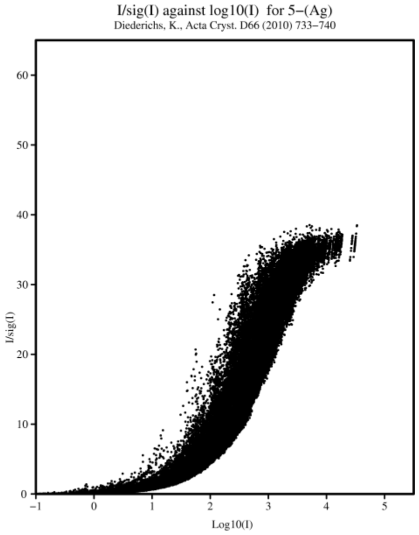 | 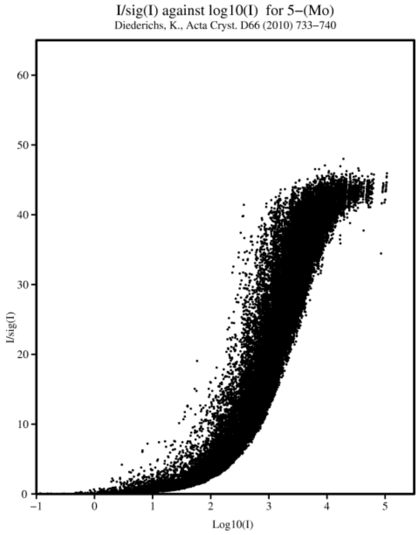 |





**Data of sample 6:**

| Ag | Mo |
|---|---|
| 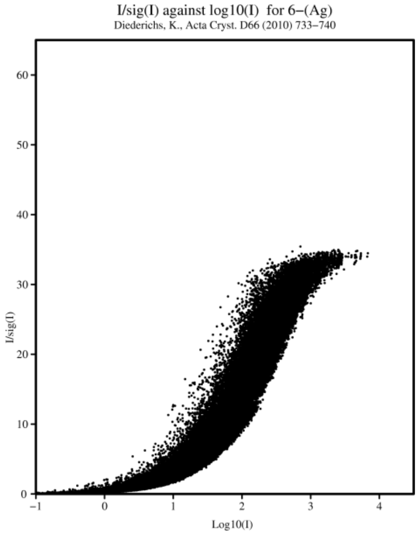 | 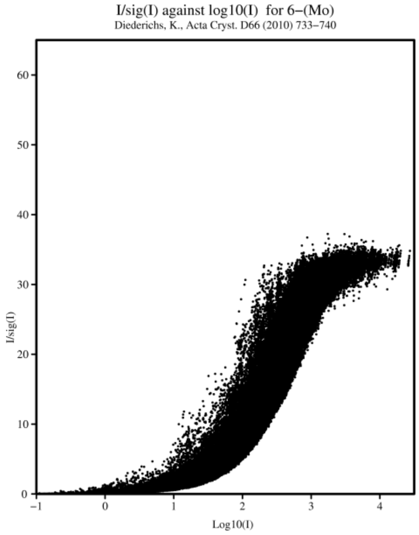 |
